# White matter measures correlate with essential tremor severity—A pilot diffusion tensor imaging study

**DOI:** 10.1002/brb3.1039

**Published:** 2018-07-02

**Authors:** Igor Nestrasil, Alena Svatkova, Kyle D. Rudser, Ravishankar Chityala, Amy Wakumoto, Bryon A. Mueller, Petr Bednařík, Paul Tuite, Xiang Wu, Khalaf Bushara

**Affiliations:** ^1^ Division of Clinical Behavioral Neuroscience Department of Pediatrics University of Minnesota Minneapolis Minnesota; ^2^ Department of Medicine III, Clinical Division of Endocrinology and Metabolism Medical University of Vienna Vienna Austria; ^3^ Multimodal and Functional Neuroimaging Research Group Central European Institute of Technology Masaryk University Brno Czech Republic; ^4^ Division of Biostatistics University of Minnesota Minneapolis Minnesota; ^5^ Minnesota Supercomputing Institute University of Minnesota Minneapolis Minnesota; ^6^ Department of Psychiatry University of Minnesota Minneapolis Minnesota; ^7^ Department of Radiology Center for Magnetic Resonance Research University of Minnesota Minneapolis Minnesota; ^8^ High Field MR Centre Department of Biomedical Imaging and Image‐guided Therapy Medical University of Vienna Vienna Austria; ^9^ Department of Neurology University of Minnesota Minneapolis Minnesota; ^10^ Psychology Department Sun Yet‐Sen University Guangzhou Guangdong China; ^11^ Neurology Service Veterans Affairs Medical Center Minneapolis Minnesota

**Keywords:** diffusion tensor imaging, essential tremor, Guillain‐Mollaret triangle, radial diffusivity, tremor network, TremScore

## Abstract

**Background:**

An evolving pathophysiological concept of essential tremor (ET) points to diffuse brain network involvement, which emphasizes the need to investigate white matter (WM) changes associated with motor symptoms of ET.

**Objectives:**

To investigate ET‐related WM changes and WM correlates of tremor severity using tremor clinical rating scales and accelerometry.

**Methods:**

Tract‐based spatial statistics (TBSS) approach was utilized to compare 3 Tesla diffusion tensor imaging (DTI) data from 12 ET patients and 10 age‐ and gender‐matched healthy individuals. Clinical scales, tremor frequency and amplitude as measured by accelerometry were correlated with DTI data.

**Results:**

ET patients demonstrated mean (MD) and radial diffusivity (RD) abnormalities in tracts involved in primary and associative motor functions such as bilateral corticospinal tracts, the superior longitudinal fascicles, and the corpus callosum but also in nonmotor regions including the inferior fronto‐occipital and longitudinal fascicles, cingulum bundles, anterior thalamic radiations, and uncinate fascicles. A combined tremor frequency and amplitude score correlated with RD and MD in extensive WM areas, which partially overlapped the regions that were associated with tremor frequency. No significant relationship was found between DTI measures and clinical rating scales scores.

**Conclusions:**

The results show that ET‐related diffusion WM changes and their correlates with tremor severity are preferentially located in the primary and associative motor areas. In contrast, a relationship between WM was not detected with clinical rating scales. Accelerometry parameters may, therefore, serve as a potentially useful clinical measures that relate to WM deficits in ET.

## 1 INTRODUCTION

Essential tremor (ET) is the most prevalent movement disorder affecting up to 5% of the general population above the age of 65 years (Louis & Ferreira, 2010), but its pathophysiology remains poorly understood. ET is characterized by a 4–12 Hz postural and kinetic tremor (Bareš, Husárová, & Lungu, 2012). For decades, studies have suggested various morphological changes underlying motor symptoms in ET (Gironell et al., 2012; Louis, Faust, & Vonsattel, 2012). However, the relatively common occurrence of nonmotor symptoms such as cognitive, behavioral, and sleep disturbances in ET led to the replacement of the long‐held “benign” mono‐symptomatic model with a complex concept of ET (Bhalsing et al., 2014). The complexity of ET‐related pathology was supported by findings of cerebellar abnormalities and widespread brain neuro‐degeneration (Louis, 2014b, 2014c). The cerebello‐thalamo‐cortical network (Sharifi, Nederveen, Booij, & van Rootselaar, 2014) (cerebello‐thalamo‐cortical [CTC], also known as “tremor network”) has been proposed to play a substantial role in ET. In CTC network, the inferior olive nuclei project, via climbing fibers, to Purkinje cells in the contralateral cerebellar cortex, which send GABAergic inhibitory projections to the dentate and other deep cerebellar nuclei that in turn project to cortical brain areas via thalamic projections and to the red nucleus (Sharifi et al., 2014).

Diffusion tensor imaging (DTI) has been increasingly utilized as a noninvasive MRI technique, which allows quantifying microstructural substrate of disease‐related white matter (WM) alterations based on water movement (Alexander, Lee, Lazar, & Field, 2007; Basser & Pierpaoli, 2011). The three‐dimensional DTI tensor model provides fractional anisotropy (FA), mean (MD), radial (RD), and axial (AD) diffusivity values (Alexander et al., 2007; Pierpaoli & Basser, 1996). FA as the degree of anisotropy varies from 0 in nondirectional to 1 in highly oriented tissue (Alexander et al., 2007) and is influenced by the degree of myelination, axonal packing and size, coherence, and co‐linearity of fiber organization (Alexander et al., 2007). Changes in cellularity, edema, and/or necrosis affect MD, while RD and AD are sensitive to membrane coherence and axonal changes; respectively (Alexander et al., 2007).

Previous DTI studies in ET confirmed an involvement of the CTC (Jia, Jia‐lin, Qin, Qing, & Yan, 2011; Klein et al., 2011; Nicoletti et al., 2010; Saini et al., 2012) but also of areas outside the “tremor network” (Klein et al., 2011; Saini et al., 2012) when a whole‐brain DTI analysis using tract‐based spatial statistics (TBSS) (Smith et al., 2006) was applied. The areas outside CTC were linked to nonmotor ET symptoms (Benito‐León et al., 2017; Bhalsing et al., 2014, 2015), which provides further evidence for the pathophysiological complexity of ET.

The widespread and variable WM alterations in ET (Jia et al., 2011; Klein et al., 2011; Nicoletti et al., 2010; Saini et al., 2012) shown in prior studies emphasize the need to further investigate the correlation of WM microstructure changes with motor signs in ET and to identify potential morphological markers of tremor severity. Identifying the relevant WM areas responsible for motor and nonmotor signs in ET would help explain the variable pharmacotherapy response in ET (Hedera, Cibulčík, & Davis, 2013) and potentially contribute to the development of reliable measures of therapeutic interventions (Ondo, 2016). Several DTI studies (Klein et al., 2011; Nicoletti et al., 2010; Pelzer et al., 2017; Saini et al., 2012) utilized clinical rating scales (e.g., Fahn‐Tolosa‐Marin [FTM] and Bain scales) as a measure of tremor severity; however, only two studies found a correlation between the FTM score and frontoparietal WM microstructure (Klein et al., 2011; Pelzer et al., 2017). The lower inter‐rater reliability and the subjective nature of the clinical rating scales (Elble et al., 2013) can be mitigated by objective accelerometry measures (i.e., the tremor frequency and amplitude), which may provide a more accurate representation of the disease pathophysiological features. This is suggested by recent functional and morphometric MRI studies (Gallea et al., 2015; Popa et al., 2013). Using MRI morphometry, Gallea and colleagues showed that the cerebellar vermis volume was uniquely correlated to tremor frequency but not to FTM clinical scale (Gallea et al., 2015). Functional MRI data also demonstrated a relationship between connectivity within CTC and accelerometry tremor measures (Gallea et al., 2015; Popa et al., 2013). Although these recent MRI studies (Gallea et al., 2015; Popa et al., 2013) do imply a link between objective tremor measures and structural brain changes within CTC, there have been no studies that specifically investigated the correlation between the WM changes in ET and tremor amplitude and frequency.

In this study, we utilized TBSS (Smith et al., 2006), as a method that allows exploring whole‐brain DTI metrics, to examine the relationship between WM changes and both clinical rating scales (i.e., FTM and Bain scale) and accelerometry tremor measures (i.e., amplitude, frequency and their combined score).

## 2 MATERIALS AND METHODS

### 2.1 Participants

Twelve patients with essential tremor (mean age 45.5 ± 17.5 years, range 19.2–75.5, 8 males/4 females, 10 right‐/1 left‐handed/1 ambidextrous) and 10 age‐ and gender‐matched controls (mean age 46.6 ± 14.8 years, range 25.5–75.15, 6 males/4 females, 8 right‐/2 left‐handed) were included in the study. The handedness was determined based on the Edinburgh Handedness Inventory (Oldfield, 1971). ET was diagnosed based on the clinical evaluation by two board‐certified neurologists specialized in movement disorders (PT, KB) using established criteria (Deuschl, Bain, & Brin, 1998). Participants had no history of other neurologic or psychiatric disorder, cognitive deficit (Mini Mental State Examination Score >25), traumatic brain injury, or seizure. All patients were off their tremor medication for 10 days prior to the study. All participants signed an informed consent according to the guidelines of the Institutional Review Board at the University of Minnesota.

Essential tremor subjects completed two clinical scales to assess tremor characteristics and severity. The Bain scale (Bain et al., 1993) evaluated severity of patient's head, voice tremors, and postural tremor of the both upper and lower limbs. The modified FTM rating scale (Elble et al., 2006) graded severity of the tremor during daily life activities, specifically finger‐ and dot‐pointing, Archimedes spiral drawing, drawing of a straight line and sine wave, handwriting, and water pouring for both hands. Physiological tremor measures were recorded in the vertical axis using a tri‐axial accelerometer, that is, Tremorometer (160‐Hz sampling rate, 2 mg resolution; FlexAble‐Systems, Fountain Hills, AZ) (Caligiuri & Tripp, 2004). A large study conducted on 242 patients with various tremor types (i.e., Parkinson's disease, ET, neuroleptic‐ and lithium‐induced tremor) confirmed ability of the Tremorometer to reliably and reproducibly generate all components of the TremScore such as amplitude, frequency, and percentage of the tremor presence. Study also confirmed clinical utility of TremScore components to differentiate tremors of distinct etiologies (Caligiuri & Tripp, 2004). In addition, Tremorometer was utilized along with MEG to assess motor cortex during voluntary and involuntary movements (Bowyer et al., 2007). In our study, the amplitude and frequency of tremor for both upper limbs were calculated during postural task while the subject was sitting with both arms extended and hands in a pronated position, parallel to the ground. The posture was maintained for 21 s, which was duration of one trial. The amplitude and the peak frequency were calculated using spectral analysis (Fast Fourier transformation) (Caligiuri & Tripp, 2004). The TremScore was automatically generated as following: weighted tremor frequency mean (WFM, in Hz), tremor amplitude per second (TA, in mg), and the tremor presence percentage (SPREAD) within a trial were combined into a single score with equal weight to all three values (TremScore, Tremorometer Reference Manual, FlexAble‐Systems, Fountain Hills, AZ).

$$\text{TremScore}=\frac{\left(\text{WFM}\times \text{TA}\times \text{SPREAD}\right)}{10}$$

The weighted frequency mean (WFM) represents variability in the tremor frequency independently of amplitude changes (Caligiuri & Tripp, 2004). Amplitude was internally converted from mm/s^2^ to the mg equivalent for calculating the TremScore. The tremor is considered more severe if the frequency is higher (WFM), its amplitude is higher (TA) or percent of time present is higher (SPREAD). The value is divided by 10 to keep the numbers in a reasonable range.

Three trials were recorded for each hand and averaged. All scores were then averaged between two hands and were examined for outliers that were excluded from analysis (Table 1).

**Table 1:** Clinical characteristics and accelerometry outcomes in essential tremor group

	*N*	Min	Max	Mean	*SEM*	*SD*
Age	12	19.24	75.47	45.48	5.05	17.48
Disease duration	12	5	29	15.42	2.21	7.67
Bain score (range 0–60)	11	3	30	9.45	2.42	8.03
FTM scale (range 0–56)	10	3	19	10.6	1.68	13.1
Spiral length	10	1.07	2.1	1.57	0.1	0.32
Frequency	12	5.19	9.23	7.39	0.39	1.34
Amplitude	11	12.1	1190	389	98.92	328.09
TremScore	12	9.5	606	244.17	69.75	241.63
Postural tremor (range 0–4)	12	1^a^	3	1.42	0.19	0.67
Kinetic tremor (range 0–4)	12	0	4	1.58	0.34	1.16

*Notes*

Variables are reported as minimum (min), maximum (max), mean, standard deviation (*SD*), and standard error of the mean (*SEM*). FTM: modified Fahn‐Tolosa‐Marin scale.

a Resting tremor present in one subject with score 1 (range 0–4).

### 2.2 Brain imaging

Brain scans were acquired on a 3T Siemens Tim Trio Scanner (Siemens Medical Solutions, Erlangen, Germany) with body coil excitation and the standard 12‐channel receive‐array Siemens head coil. Diffusion weighted scans were obtained with 30 directions (*b* = 1,000 s/mm^2^) and 6 un‐weighted b0 scans (*b* = 0 s/mm^2^), NEX = 2, TR (repetition time) = 8,800 ms, TE (echo time) = 90 ms, flip angle = 90°, FOV (field‐of‐view) = 256 × 256, voxel size 2 × 2 × 2 mm, 64 slices, no gap. Phase and magnitude field maps were acquired using the same voxel size and FOV as diffusion weighted scans, with TR = 700 ms, flip angle = 90°, and TE = 7.08 ms (phase image)/TE = 4.62 ms (magnitude image).

### 2.3 DTI data analysis

Diffusion tensor imaging postprocessing was performed using the FSL (Jenkinson, Beckmann, Behrens, Woolrich, & Smith, 2012) software version 5.0.6 (FMRIB Software Library, http://www.fmrib.ox.ac.uk/fsl). Fieldmaps were used to diminish EPI distortion caused by inhomogeneities in applied magnetic field. Diffusion weighted data were corrected for movement and eddy current (ECC) distortions (Smith, 2002). Average motion parameters in the *x*‐, *y*‐, *z*‐axis were calculated for each subject.

Preprocessed images were fitted to the tensor model at each voxel to generate FA, MD, AD, and RD images using DTIFIT in FSL (Jenkinson et al., 2012). TBSS (Smith et al., 2006) was chosen as an optimal full‐automated analysis tool that allows investigating the whole‐brain without selection of specific WM region beforehand. All FA images were registered into a common space using nonlinear registration with FMRIB58_FA as a registration target image (Andersson, Jenkinson, & Smith, 2007a, 2007b). Next, the transformed images were averaged to create a mean FA image. Thinning was applied to generate a study‐specific skeleton that consists of voxels representing relevant tract centers. To exclude gray matter and CSF containing voxels only skeleton voxels with an FA value larger than 0.3 were considered. Finally, FA skeletons were computed for each individual image and aligned with the study‐specific FA skeleton. Normalization warps were applied to MD, AD and RD maps. This allowed for a point‐by‐point comparison between all subjects for each of the individual skeleton voxels without preselecting specific region beforehand.

### 2.4 Statistical analysis

The exploratory analysis in SPSS 22.0 depicted two subjects with outlier modified FTM scale values. Also, one outlier for amplitude and Bain score were detected, while no outlier values were found for age, duration of illness, spiral length, tremor frequency or TremScore. Clinical and symptom severity characteristics of ET patients were summarized without outliers with minimum, maximum values, means, standard deviations, and standard errors of the means using SPSS 22.0 (Table 1). In addition, 4 patients exhibited head tremor (severity of head tremor 4.75 ± 2.06). Motion parameters between healthy subjects and ET patients as well as between patients with and without head tremor were compared using t‐test in SPSS.

The nonparametric voxel‐wise statistics in “randomize” program in FSL was used to calculate differences between patients and controls adjusted for age using threshold‐free cluster enhancement (Smith & Nichols, 2009).

Regression analyses in “randomize” were conducted to estimate role of both types of clinical scores and tremor amplitude, frequency of tremor, and the TremScore on diffusion measurements in patients exclusively with age as a covariate. In case of outlier value presence, regression analyses were conducted both with and without subjects who exhibited outlier scores for particular scale. The number of permutations was set to 5,000 for both cross‐sectional and regression analyses (Nichols & Holmes, 2002; Winkler, Ridgway, Webster, Smith, & Nichols, 2014). Results were corrected for multiple comparisons. The family‐wise error corrected *p‐*value threshold was set to <0.05. The association between significant DTI clusters and tremor severity measures was characterized by correlation coefficients and *p*‐values using FSL (Table 3). Since age has a critical confounding effect on both DTI (Salat et al., 2009) and clinical tremor measures (Elble, Higgins, Leffler, & Hughes, 1994), all regression tests were adjusted for age.

## 3 RESULTS

### 3.1 Cross‐sectional DTI differences

Parameters characterizing movement during the scanning did not significantly differ between patients and controls or between patients with and without presence of head tremor.

**Figure 1:**
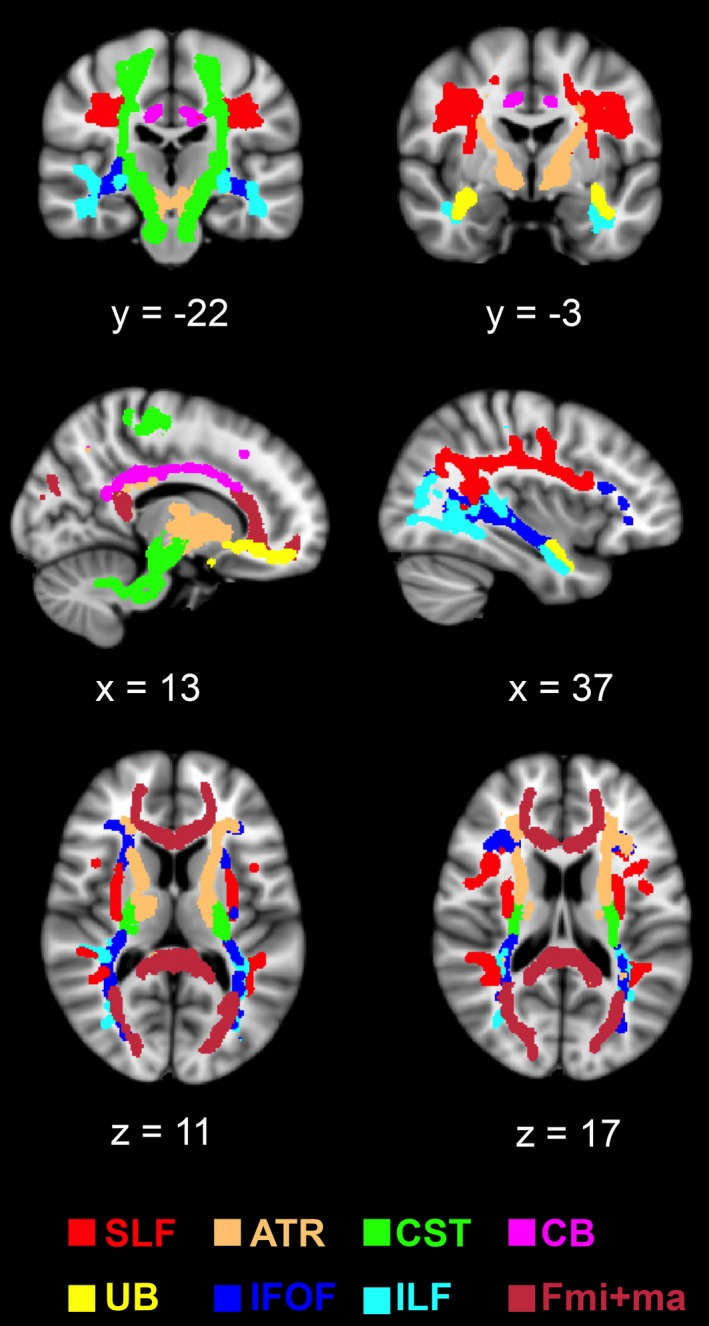
Anatomical localization the major white matter brain tracts based on the JHU‐ICBM‐tract atlas (The John Hopkins University—The International Consortium for Brain Mapping), ATR: anterior thalamic radiation; CB: cingulum bundle; CST: corticospinal tract; Fmi+ma: Forceps minor+major—Fmi is located ventrally, Fma dorsally; IFOF: inferior fronto‐occipital fascicle; ILF: inferior longitudinal fascicle; SLF: superior longitudinal fascicle; UB: uncinate bundle

**Figure 2:**
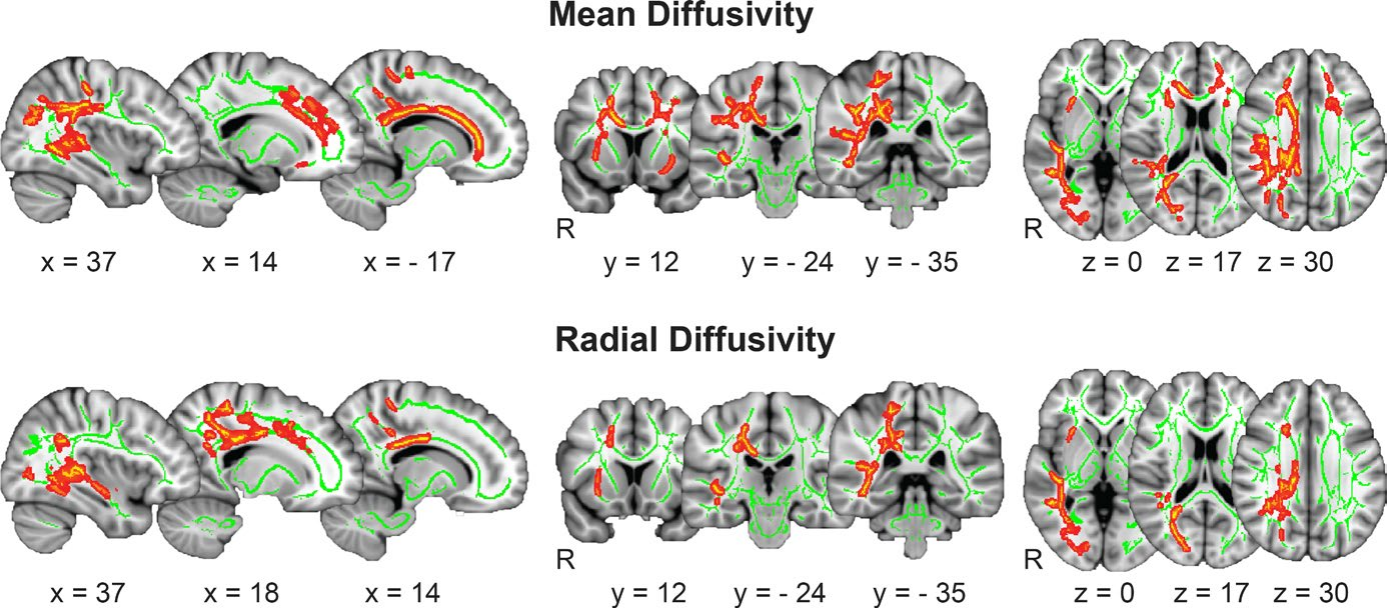
White matter differences between essential tremor patients and healthy controls. Mean (MD) and radial diffusivity (RD) differences between groups based on a voxel‐vise comparison of skeleton voxels using tract‐based spatial statistics (TBSS) within FSL. Clusters with significantly higher MD and RD in patients with essential tremor compared to healthy controls are in red‐yellow (*p*_FWEcorr_ < 0.05), voxels that belong to TBSS‐skeleton in green. *x*,* y*,* z* values are showing MNI coordinates of selected slice. Significant voxels were spatially smoothed using “fill” tool in TBSS to enhance visualization of the results

Diffusion tensor imaging analysis demonstrated significantly higher MD in ET patients compared to healthy controls in widespread WM areas (*p*_FWEcorr_ = 0.012, total number of voxels 11,422) located in the forceps minor (Fmi) and major (Fma), in the right corticospinal tract (CST), in the right inferior fronto‐occipital fascicles (IFOF), in the right superior longitudinal fascicle (SLF), in the right inferior longitudinal fascicle (ILF), in bilateral uncinate fascicles (UF), cingulum bundles (CB), and in bilateral anterior thalamic radiations (ATR). These changes were underlain by higher AD in ET compared to healthy controls in the subtle area within the Fmi (*p*_FWEcorr_ = 0.049, number of voxels 4), but mostly by significantly higher RD in the ET patients (*p*_FWEcorr_ = 0.028, number of voxels 5,348). The pathologically higher RD was found in the Fmi and Fma, the right CST, SLF, and ILF, in the right IFOF, UF, and ATR in the patients with respect to healthy individuals (Table 2, Figures 1 and 2).

**Table 2:** White matter clusters with significant differences between groups

Difference		*N*	JHU‐ICBM‐tract atlas	*p* value	MNI coordinates		
					*X*	*Y*	*Z*
MD	ET > controls	11,422	Right CST, Fmi, Fma, right IFOF, right SLF, right ILF, bilateral UF, ATR and CB	0.012	17	−37	35
AD	ET > controls	4	Fmi	0.049	11	32	6
RD	ET > controls	5,348	Right CST, Fmi, Fma, right SLF, right IFOF, right ILF, right ATR, right UF	0.028	21	−27	39

*Notes*

Number of voxels, localization based on JHU‐ICBM‐tract atlas (The John Hopkins University—The International Consortium for Brain Mapping), and corrected *p*‐values (*p*_FWEcorr_ < 0.05) for each significant cluster as revealed by threshold‐free cluster enhancement (TFCE) method in the “randomize” program part of FSL. Analyses were adjusted for age. MNI coordinates describe the localization of maximum intensity voxels in specific white matter areas.

AD: axial diffusivity; ATR: anterior thalamic radiation; CB: cingulum bundle; CST: corticospinal tract; Fma: forceps major; Fmi: forceps minor; IFOF: inferior fronto‐occipital fascicle; ILF: inferior longitudinal fascicle; MD: mean diffusivity; RD: radial diffusivity; SLF: superior longitudinal fascicle; UF: uncinate fascicle.

### 3.2 Association between DTI and tremor severity parameters

**Figure 3:**
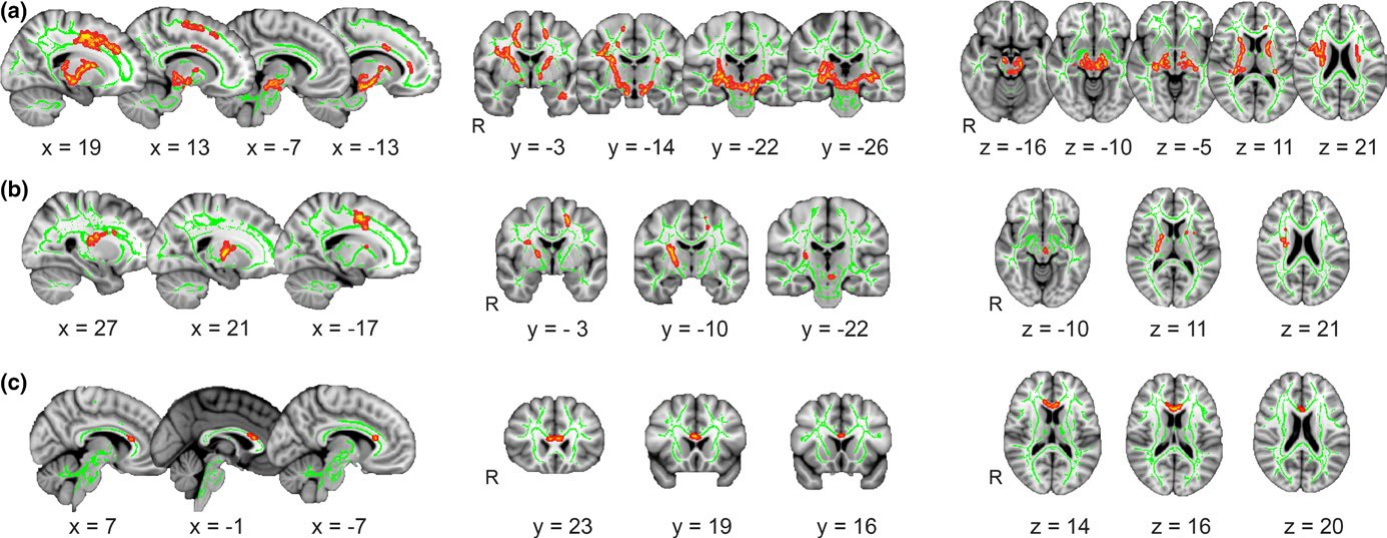
Associations between clinical characteristics and diffusion metrics. Clusters with significant associations with tremor severity in patients with essential tremor are shown in red‐yellow (*p*_FWEcorr_ < 0.05), voxels that belong to tract‐based spatial statistics (TBSS)‐skeleton in green. Significant voxels were spatially smoothed using “fill” tool in TBSS to enhance visualization of the results. (a) Mean diffusivity areas related to the TremScore. (b) Radial diffusivity values associated with TremScore. (c) Relationship between frequency and radial diffusivity

**Figure 4:**
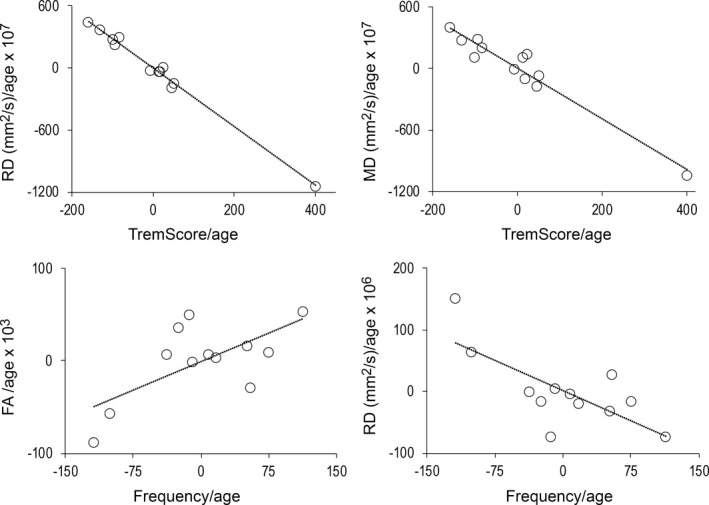
Correlation graphs. Relationship between significant clusters and clinical accelerometry parameters, all values were accounted for age (Note that the averages across the voxels used for the figure were calculated from significant clusters, thus no new *p*‐values should be calculated based on the figure)

Voxel‐by‐voxel TBSS analysis adjusted for age demonstrated a significant negative association between TremScore and MD in WM clusters (*p*_FWEcorr_ = 0.011, *R* = 0.313, 6,171 voxels) located bilaterally in SLF, CST, IFOF, ATR, UF and in the left ILF, left CB as well as Fmi. These changes were accompanied by RD (*p*_FWEcorr_ = 0.011, *R* = 0.313, 684 voxels) changes related to the same score in bilateral CST, SLF and ATR and the Fmi (Figures 3 and 4, Table 3).

**Table 3:** White matter clusters showing significant association with objective tremor measurements

	Correlation		*N*	JHU‐ICBM‐tract atlas	*R* value	*p* value	MNI coordinates		
							*X*	*Y*	*Z*
MD	TremScore	Negative	6,171	Bilateral SLF, CST, IFOF, ATR, Fmi, Left UF, left CB	0.313	0.011	27	−25	−4
RD	TremScore	Negative	684	Bilateral CST, ATR, SLF, Fmi	0.313	0.011	21	−11	7
RD	Tremor frequency	Negative	92	Fmi	0.303	0.045	2	19	18
FA	Tremor frequency	Positive	2	Left capsula externa	0.302	0.049	−30	10	−6

*Notes*

Number of voxels, localization based on JHU‐ICBM‐tract atlas (The John Hopkins University—The International Consortium for Brain Mapping), correlation coefficients and corrected *p*‐values (*p*_FWEcorr_ < 0.05) for each significant cluster as revealed by threshold‐free cluster enhancement (TFCE) method in the “randomize” program part of FSL. Analyses were adjusted for age. MNI coordinates describe the localization of maximum intensity voxels in specific white matter areas.

ATR: anterior thalamic radiation; CB: cingulum bundle; CST: corticospinal tract; FA: fractional anisotropy; Fmi: forceps minor; IFOF: inferior fronto‐occipital fascicle; MD: mean diffusivity; RD: radial diffusivity; SLF: superior longitudinal fascicle; UF: uncinate fascicle.

The modified FTM tremor rating scores showed significant positive association with AD in clusters (*p*_FWEcorr_ = 0.032, *R* = 0.307, 145 voxels) located within the left SLF, ILF, and IFOF. The relationship lost significance after exclusion of two subjects with outlier FTM values (*p*_FWEcorr_ = 0.26, *R* = 0.261).

Primary analysis adjusted for age also confirmed a significant positive correlation between frequency and FA in the left capsula externa (*p*_FWEcorr_ = 0.049, *R* = 0.302, 2 voxels) and the negative relationship with RD in the Fmi (*p*_FWEcorr_ = 0.045, *R* = 0.303, 92 voxels).

## 4 DISCUSSION

Our data show significant correlations between objective tremor measures (combined TremScore, tremor frequency) and diffusivity metrics primarily in the WM regions that are considered parts of the CTC network but also in areas beyond the tremor network. Higher MD and RD diffusivities in ET patients compared to healthy controls were found in extensive WM areas. In addition, subtle AD changes were detected in the forceps minor.

The extent and right‐sided preference of ET‐related changes are in agreement with a prior TBSS‐based DTI study (Saini et al., 2012). Given the right‐handedness of most of the participants (82%), the dominance of contralateral (left) hemisphere is expected (Gut et al., 2007). Therefore, the WM in the left hemisphere, being the substrate for a training‐induced plasticity, may be less affected by the disease (Saini et al., 2012; Scholz, Klein, Behrens, & Johansen‐Berg, 2009; Svatkova et al., 2015). While the contribution of both AD and RD on the MD changes point to the complex nature of WM changes with alterations in water content, myelin or axonal structure(Alexander et al., 2007; Saini et al., 2012), more widespread MD disturbance mostly contributed to RD abnormalities implies an abnormal myelination (Song et al., 2002) in the ET pathology. Similar to prior studies, we did not detect significant changes in FA, while demonstrating differences in diffusivity measures (Saini et al., 2012). Although FA represents a very robust WM measurement (Basser & Pierpaoli, 2011), it specifically shows a relative variation between the levels of diffusion measured in different direction (Alexander et al., 2007; Basser & Pierpaoli, 2011). FA may remain relatively stable even if individual diffusivity values undergo relatively large changes.

White matter areas related to objective tremor measures as well as changes detected in patients compared to controls were anatomically located within the same tracts associated preferentially with motor functions. ET‐related deficits were demonstrated in the CST, the primary motor projection of the cortical motor areas to the spinal cord (Martin, 2005). WM alteration within CST in ET has been demonstrated previously (Gallea et al., 2015; Saini et al., 2012) and corroborate with functional MRI studies showing altered brain activity in motor cortical areas at rest (Fang et al., 2016), during the grip‐force (Neely et al., 2015) and finger‐tapping task in ET patients (Buijink, Broersma, et al., 2015). Similar findings were also shown with complex motor tasks such as extension of the right hand and arm to induce tremor (Buijink, van der Stouwe, et al., 2015). Importantly, WM changes in CST correlated with physiological characteristics of tremor, reported as a complex value composed of tremor frequency, amplitude, and the percentage of tremor presence (i.e., TremScore). Although patients demonstrated higher MD and RD compared to healthy individuals, objective tremor measures showed inverse relationship with these metrics when age was taken into account. Thus, younger patients with high scores exhibited lower (“better”) RD than older patients with similar tremor score values and vice versa. This points to the progressiveness of ET‐related WM changes and further supports the proposed neurodegenerative nature of ET (Louis, 2014b). Also, ET in younger versus elder patients can have a different disease nature supporting the hypothesis of ET being not a single disease but rather a family of diseases with a large heterogeneity (Louis, 2014a). The lower RD values, are often associated with higher myelination (Song et al., 2002), which might lead to increased transmission velocity between brain regions but does not necessarily indicate an increased communication efficiency (Laughlin & Sejnowski, 2003) and may, in fact, represent a compensation for aberrant function elsewhere in the brain (Mandl et al., 2010). While patients demonstrated changes suggestive of lower myelination levels compared to controls, the opposite correlation between tremor score in patients group suggests a compensatory increase in structural connectivity to balance clinical deficits. Importantly, areas that correlate with objective tremor measures extend to the midbrain, specifically areas around the cerebral peduncles and red nucleus thus including the WM within dentate‐rubro‐olivary pathway (Guillain‐Mollaret triangle), which is considered a tremorgenic loop (Murdoch, Shah, & Jampana, 2016). These findings are consistent with previous studies showing abnormalities in DTI parameters directly within red nucleus (Jia et al., 2011), in retrorubral WM (Shin, Han, Kim, & Lee, 2008) and the Guillain‐Mollaret triangle (Nicoletti et al., 2010) in ET patients. However, no correlation with tremor clinical parameters was reported in these studies (Jia et al., 2011; Nicoletti et al., 2010; Shin et al., 2008). Our data show a unique correlation of CST changes exclusively with combined TremScore but not with frequency or FTM scale. Similarly, forceps minor located in the ventral part of the corpus callosum showed a significant correlation with both objective combined score and frequency but not the clinical rating scales. While the absence of correlation between diffusion measurements and clinical ET rating scales in our study corroborates two previous DTI studies conducted on the larger sample of 22 and 25 ET patients (Nicoletti et al., 2010; Saini et al., 2012), others showed significant association between frontoparietal WM and Fahn score in smaller samples of 14 and 19 ET patients, respectively (Klein et al., 2011; Pelzer et al., 2017). These discrepancies may be explained by lower inter‐rater reliability and the subjective nature of the clinical rating scales (Elble et al., 2013). We cannot exclude that the correlation with clinical rating scales could be detected on larger ET populations, although our sample shows significant relationships between WM and objective tremor measures but no association with clinical scales after exclusion of outliers and thus suggests that objective tremor measures reflect WM deficits.

The different association patterns with WM changes of tremor frequency and amplitude suggests that tremor frequency and amplitude are generated by different mechanisms involving different brain structures which may explain our finding that combined score was more extensively reflected in WM than frequency per se, while amplitude alone failed to show any significant correlations.

White matter alteration in ET compared to control group, as well as association with objective measures, were also reported in the SLF that is partially involved in motor functions (Makris et al., 2005). The SLF represents a complex fiber bundle that facilitates executive functioning but also encodes the position of the body in the surrounding space and monitors complex motor activities (Makris et al., 2005). Thus, our data provide compelling evidence of an involvement of primary and associative motor tracts in ET.

In addition, our data also shown WM changes in ET patients within fiber bundles such as ATR, IFOF, ILF, and UF as well as CB. WM microstructure in abovementioned bundles was related to various neuropsychological outcomes in ET (Bhalsing et al., 2015), while our study linked WM blobs within these tracts to objective tremor measurements reflecting abnormal motor functions. These relationships were in partial agreement with an association between FTM scale and fronto‐parietal WM deficits demonstrated previously (Klein et al., 2011). In ATR (Mamah et al., 2010), which spreads from thalamus to frontal regions, were the tremor‐related areas located directly in the thalamus. Indeed, previous PET studies showed increased thalamic blood flow during rest (Wills, Jenkins, Thompson, Findley, & Brooks, 1994) and passive movement performance (Jenkins et al., 1993; Wills, Jenkins, Thompson, Findley, & Brooks, 1995) in ET patients and the stereotactic surgery of ventral intermediate nucleus of the thalamus has been proved an efficient treatment of ET (Flora, Della Flora, Perera, Cameron, & Maddern, 2010). Thus, an association with physiological tremor characteristics found in our studies further substantiates the importance of thalamic connections in ET symptomatology. At this point, we might only speculate about relationships between tremor symptoms and WM clusters located in UF, ILF, and IFOF. All these bundles are involved in memory retrieval, reward processing, emotional regulation, impulsive responding, semantic language processing, attention, and reversal learning (Mandonnet, Nouet, Gatignol, Capelle, & Duffau, 2007; Martino & De Lucas, 2014; Olson, Von Der Heide, Alm, & Vyas, 2015), although orbitofrontal WM, part of both UF and ILF, was associated with the FTM score (Klein et al., 2011) and showed affected in previous ET study (Shin et al., 2008). Also, atrophy in frontal and temporal gray matter regions, which are interconnected by UF, was found related to the FTM scale (Bagepally et al., 2012) and an improvement of WM integrity within the ILF and IFOF was consistently reported among longitudinal DTI studies evaluating effects of physical training (Huang, Lu, Song, & Wang, 2015; Scholz et al., 2009), implying their involvement in motor functions. However, the absence of comprehensive psychological evaluations does not allow us to rule out the possibility that WM abnormalities detected in ET patients are to certain level related to psychological deficits as published previously (Benito‐León et al., 2017; Bhalsing et al., 2015). Future studies with particular focus on the examination of different impacts of both cognitive and motor signs on WM substrate are certainly warranted.

Contrary to one whole‐brain DTI study (Saini et al., 2012) but in agreement with another (Klein et al., 2011), we did not find any significant changes in the cerebellum or cerebellar peduncles using TBSS method (Smith et al., 2006). Klein et al. (2011) suggested that differences between region‐of‐interest (ROI) and whole‐brain methods are explicitly in the cerebellar peduncles caused by an inability of the FA‐driven registration techniques to properly align these structures, particularly to distinguish inferior and medial cerebellar peduncles. While TBSS is more suitable for exploratory studies as it does not exclude any region beforehand, the stringent multiple comparisons used for our statistical analysis (Smith & Nichols, 2009) might eliminate results potentially revealed by ROI analyses. Indeed, Klein et al. (2011) did not report cerebellar changes using whole‐brain analysis but confirmed an involvement of cerebellar peduncles using ROI analysis method.

## 5 LIMITATIONS

The small number of subjects is the main limitation of this study. Further studies are needed to confirm our findings. Small sample also did not allow us to separate subjects based on a family history of ET, response to alcohol, head or resting tremor presence. Since our study did not provide a comprehensive neuropsychological assessment, we cannot evaluate the relationship between cognition and WM changes and completely exclude the possibility that WM alteration is to certain level caused by cognitive deficits.

## 6 CONCLUSIONS

Our results showed widespread WM alterations in ET patients, which further supports the neurodegenerative nature of the disease. Accelerometry tremor parameters showed robust correlation with changes in WM of the primary and associative motor areas, which were not detectable with clinical rating scales. Objective tremor metrics may therefore serve as important clinical measurements that correlate with WM alterations in ET.

## CONFLICT OF INTERESTS

The authors report no conflict of interests.
